# Antioxidant and Anti-Inflammatory Activities in Relation to the Flavonoids Composition of Pepper (*Capsicum annuum* L.)

**DOI:** 10.3390/antiox9100986

**Published:** 2020-10-13

**Authors:** Soo-Yeon Cho, Heon-Woong Kim, Min-Ki Lee, Hyeon-Jung Kim, Jung-Bong Kim, Jeong-Sook Choe, Young-Min Lee, Hwan-Hee Jang

**Affiliations:** 1 Functional Food Division, National Institute of Agricultural Sciences, Rural Development Administration, Wanju 55365, Korea; 0727jsy@korea.kr (S.-Y.C.); ksharrier@korea.kr (H.-W.K.); mklove22@naver.com (M.-K.L.); khj0913@korea.kr (H.-J.K.); jungbkim@korea.kr (J.-B.K.); swany@korea.kr (J.-S.C.); 2 Division of Applied Food System, Major of Food and Nutrition, Seoul Women’s University, Seoul 01797, Korea

**Keywords:** pepper leaf, pepper fruit, luteolin, reactive oxygen species

## Abstract

The chili pepper (*Capsicum annuum* L.) is a food source that is rich in flavonoids such as luteolin and apigenin. Flavonoids are known to have anti-inflammatory and antioxidant activities; however, studies on the flavonoids composition identified and the anti-inflammatory and antioxidant effects in pepper leaves (PL) and fruits (PF) are insufficient. In the present study, we investigated the antioxidant and anti-inflammatory effects in vitro, and the flavonoids contents of the PL and PF. Pepper extracts showed radical scavenging activities and ameliorated the lipopolysaccharide (LPS)-stimulated inflammatory response by decreasing nitric oxide production and interluekin-6 and tumor necrosis factor alpha levels in RAW 264.7 cells, with more effective activities noted for PL than for PF. Furthermore, PL extracts markedly inhibited the LPS-induced production of reactive oxygen species accumulation. The flavonoid profile and content of pepper were dependent on the part, with PL showing higher total flavonoids than PF. In particular, the content of luteolin glycosides in PL was twice that in PF. Thus, PL may be useful to prevent oxidative stress and inflammation-related diseases.

## 1. Introduction

The chili pepper (*Capsicum annuum* L.), a perennial herbaceous plant belonging to the Solanaceae family, is a widely consumed vegetable worldwide, including in Asia. In addition, pepper is a commonly used pungent spice, including as a valuable ingredient in kimchi, a traditional Korean food. Spices have increasingly attracted attention with the recognition of their beneficial effects for human health [1,2]. Although pepper fruits (PF) are mostly used as vegetable foods or spices, pepper leaves (PL) are also consumed as cooked vegetables in Korea. 

*C. annuum* has been reported to be an excellent source of antioxidants such as vitamin C and carotenoids [3,4]. The capsaicinoids, which are responsible for the pungency of pepper, are characterized by chemopreventive, antioxidant, anti-inflammatory, and weight-reducing effects [5,6]. Peppers are also a good source of phenolic compounds, particularly flavonoids [3,7,8]; however, the detailed flavonoids composition identified in PL and PF is insufficient. 

Flavonoids have been shown to have beneficial effects on the prevention of various chronic diseases, including cancers, cardiovascular diseases, and type 2 diabetes, which is attributed principally to their anti-inflammatory and antioxidant effects [9,10,11]. A meta-analysis of cohort studies indicated that dietary total flavonoids consumption reduced all-cause mortality [12]. As a strong dietary source of flavonoids, pepper has been claimed to show a variety of biological activities, including anti-inflammatory and antioxidant effects. However, the anti-inflammatory and antioxidant effects of *C. annuum* have not been reported in detail, especially according to the different parts of the plant. 

Therefore, in the present study, we aimed to investigate the antioxidant and anti-inflammatory effects of PL and PF in vitro, along with the respective flavonoids contents of each part using ultra high-performance liquid chromatography (UPLC)–diode array detector (DAD)–quadrupole time-of-flight (QToF)–mass spectrometry (MS). We hypothesized that *C. annuum* ethanol extracts would have flavonoids-derived antioxidant and anti-inflammatory effects, which would differ for the leaves and fruits. 

## 2. Materials and Methods 

### 2.1. Sample Preparation

PL and PF samples, which are immature green hot peppers, were collected from a farm in Yecheon, the northeastern region of Korea. After cleaning and drying, the samples were crushed using a grinder and sealed in a plastic bag. Then, 30 g of each powder were extracted twice with 300 mL of 80% ethanol at 25 °C for 19 h. The extracts of PL and PF were filtered through No. 6 filter paper (Advantec Co., Tokyo, Japan) and concentrated to dryness using a rotary evaporator (EYELA N-1000; Riakikai Co., Ltd., Tokyo, Japan) at 37 °C. The filtrates were then frozen, lyophilized, and stored until further use.

### 2.2. Determination of 1,1-Diphenyl-2-Picrylhydrazyl (DPPH) Radical Scavenging Activity

The DPPH radical scavenging activity test was conducted according to a previously described method [13], with slight modifications, and was performed in triplicate. PL and PF ethanol extracts (10 mM in ethyl alcohol) were diluted with phosphate-buffered saline (PBS) for the in vitro assay. Then, 100 µL of each extract (100, 200, and 500 µg/mL) were mixed with 100 µL DPPH reagent (1.5 × 10^−4^ M, Sigma-Aldrich Co.). After 30 min incubation in the dark at 25 °C, the absorbance was measured at 518 nm using luteolin and ascorbic acid as the positive control. The antioxidant activity was calculated using the following formula:

Radical scavenging activity (%) = [(Ac − As)/Ac] × 100,

where “As” and “Ac” represented absorbance values of the sample and control solution, respectively.

### 2.3. 2,2′-Azino-Bis(3-Ethylbenzothiazoline-6-Sulfonic Acid (ABTS·^+^) Radical Scavenging Activity

The ABTS·^+^ assay was performed in triplicate following previously published methods [14]. First, 10 mL of 7.4 mM ABTS stock solution were mixed with 10 mL of 2.6 mM potassium persulfate and left in the dark for 15 h. Next, the resultant ABTS·^+^ solution was diluted to 1.5 absorbance at 734 nm, and 50 μL of each PL and PF extract (100, 200, and 500 µg/mL) were mixed with 150 µL ABTS·^+^ solution. After 15 min of incubation at room temperature, the absorbance was measured at 734 nm with luteolin and ascorbic acid as the positive control. The antioxidant activity was calculated using the same formula as described above for the DPPH test in Section 2.2.

### 2.4. Cells and Culture

The RAW 264.7 murine macrophage cell line was obtained from the Korea Cell Line Bank (Seoul, Korea). The cells were cultured in Dulbecco’s modified Eagle’s medium (Gibco, Rockville, IL, USA) supplemented with 10% heat-inactivated fetal bovine serum (Gibco) and penicillin–streptomycin solution (100 U/mL penicillin and 100 µg/mL streptomycin; Hyclone Laboratories Inc., South Logan, UT, USA). The cells were grown in a 5% CO_2_ humidified atmosphere at 37 °C.

### 2.5. Cell Viability

Cell viability was assessed using the MTS assay. RAW 264.7 cells were plated at a density of 2 × 10^5^ cells/well in a 96-well plate. After being cultured in the presence of 100 μg/mL, 200 μg/mL, and 500 μg/mL PF and PL extracts for 18 h, the cells were subjected to the viability test using the CellTiter 96 Aqueous One Solution Cell Proliferation Assay kit (Promega, Fitchburg, WI, USA) following the manufacturer’s manual. Each test was performed in triplicate.

### 2.6. Determination of Nitric Oxide (NO) Production 

RAW 264.7 cells were plated at a density of 2 × 10^5^ cells/well in a 96-well plate. The cells were incubated with 100 ng/mL lipopolysaccharide (LPS) in the presence of ethanol extracts of PL or PF (100, 200, and 500 µg/mL) for 18 h. After incubation, culture supernatants (100 µL) were mixed with an equal volume of Griess reagent and allowed to react for 15 min. Then, the absorbance was measured at 540 nm using a microplate reader (Molecular Devices Inc., Sunnyvale, CA, USA). The nitrite concentration in the sample was calculated using a standard curve prepared with NaNO_2_.

### 2.7. Determination of Interleukin (IL)-6 and Tumor Necrosis Factor (TNF)-α

RAW 264.7 cells (2 × 10^5^ cells/well) were incubated with 100 ng/mL LPS in the presence of ethanol extracts of PL or PF (100, 200, and 500 µg/mL) for 18 h. After 18 h, the culture supernatants were collected, and the levels of IL-6 and TNF-α in the culture media of RAW 264.7 cells were determined using an enzyme-linked immunosorbent assay kit (R&D Systems, Minneapolis, MN, USA) according to the manufacturer’s protocol. The IL-6 and TNF-α levels were determined using a microplate reader.

### 2.8. Determination of Reactive Oxygen Species (ROS)

The production of ROS was detected using fluorescence microscopy with dichloro-dihydro-fluorescein diacetate (DCFH_2_-DA). Briefly, RAW 264.7 cells (1 × 10^5^ cells/well) were incubated with 100 ng/mL LPS in the presence of ethanol extracts of PL and PF (100 and 500 µg/mL), and luteolin (10 and 50 µM) as positive control for 18 h. Then, 50 µM DCFH_2_-DA was added to the culture medium, and the cells were incubated at 37 °C for an additional 30 min. After incubation, the cells were washed with warm PBS. The DCF fluorescence was measured using fluorescence microscopy (ZEISS Axiovert 40 CFL, Oberkochen, Germany) at 100× magnification.

### 2.9. Qualitative and Quantitative Analysis of Flavonoids Using UPLC-DAD/QTOF-MS

Powdered PF (1 g) and PL (0.1 g) were extracted with 10 mL of methanol:water:formic acid (50:45:5, *v/v/v*) including an internal standard solution (galangin, 20 ppm) for 10 min at room temperature in a shaker, and then centrifuged (900× *g*, 10 min, 10 °C). The supernatant was passed through a 0.2 µm syringe filter (Whatman International Ltd., Maidstone, Kent, UK), and then the filtrate was analyzed using UPLC–DAD–QToF–MS. Standards of quercetin 3-O-glucoside (isoquercitrin), quercetin 3-O-rhamnoside (quercitrin), luteolin 8-C-glucoside (orientin), luteolin 6-C-glucoside (isoorientin), apigenin 7-O-glucoside (comsosiin), apigenin 6-C-glucoside-8-C-arabinoside (schaftoside), and apigenin 6-C-arabinoside-8-C-glucoside (isoschaftoside) were purchased from Extrasynthese (Genay Cedex, France). The flavonoids were identified and quantified using an ACQUITY UPLC–DAD system (Waters Co., Milford, MA, USA) and a SYNAPT mass spectrometer (Waters Micromass, Manchester, UK). In addition, a Kinetex XB-C18 100A column (column internal diameter is 2.1 mm, length is 150 mm, and particle size is 1.7; Phenomenex Inc., Torrance, CA, USA) was used. The analysis was conducted at a flow rate of 0.3 mL/min and detection wavelength of 210–400 nm (a representative wavelength of 350 nm) in a 30 °C oven. The mobile phases used were 0.1% formic acid in water (phase A) and 0.1% formic acid in acetonitrile (phase B). The pretreated sample was analyzed using the following gradient: 5–25% B from 0 to 20 min; 25–50% B for 5 min, 50–90% B for 5 min, 90% B for 2 min, and 90–95% B for 5 min, followed by a final wash with 5% B for 5 min. The MS analysis was run in positive ionization mode using an electrospray ionization source. The MS parameters were set to a sampling cone voltage of 40 V, capillary voltage of 3.5 kV, source temperature of 120 °C, desolvation temperature of 500 °C, and desolvation N_2_ gas flow rate of 1050 L/h. The range of molecular weights was m/z 200–1200 in full scan mode.

### 2.10. Statistical Analysis

The data are presented as the mean ± standard deviation of three individual experiments performed in triplicate. All statistical analyses were performed using SAS 9.2 (SAS Institute, Cary, NY, USA). The results were analyzed using a one-way analysis of variance. When a significant difference was indicated, Duncan’s multiple-range test was performed to determine significant differences among the groups. Statistical significance was based on the difference compared with CON (+) and was judged at *p* < 0.05.

## 3. Results

### 3.1. Radical Scavenging Activity of the PL and PF Extracts 

Table 1 shows the results of the DPPH and ABTS assays on the PL and PF extracts. The half-maximal effective concentration (EC_50_) for DPPH radical scavenging activity was 2.1 µg/mL in luteolin, which was lower than 6.4 µg/mL in ascorbic acid. The EC_50_ of PL was 159.1 µg/mL, which was lower than that of the PF with EC_50_ of 653.4 µg/mL. The EC_50_ for the ABTS radical scavenging activity was also lower in PL than in PF (157.6 vs. 357.1 µg/mL). Therefore, PL showed stronger antioxidant activity than PF. Treatment with a high concentration (500 µg/mL) of PL showed more than 90% scavenging activity of DPPH and ABTS radicals.

### 3.2. Effect of the PL and PF Extracts on Inflammation

**Figure 1:**
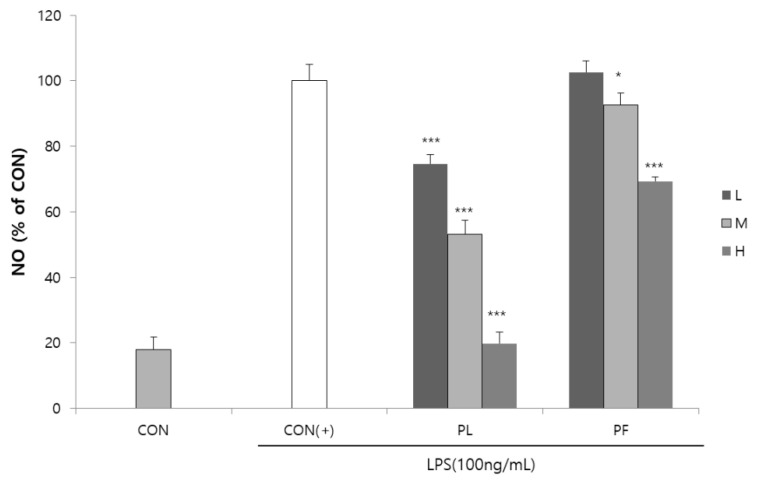
Inhibition of nitric oxide (NO) production by the extracts of pepper leaves (PL) and pepper fruits (PF) in lipopolysaccharide (LPS)-stimulated RAW 264.7 cells. RAW 264.7 cells were treated with 100 (L), 200 (M), and 500 (H) µg/mL PL and PF extracts in the presence of 100 ng/mL LPS for 18 h. Statistical significance is based on the difference compared with CON(+) (* *p* < 0.05, *** *p* < 0.001).

**Figure 2:**
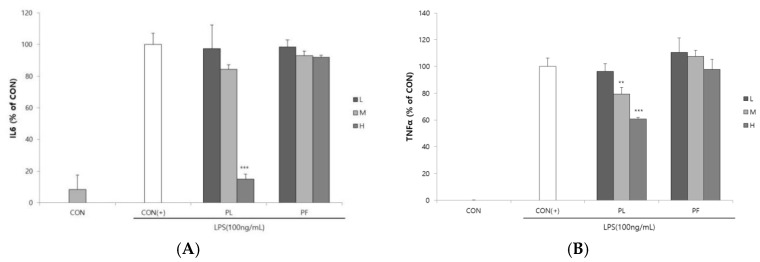
Suppression of (**A**) interleukin (IL)-6 and (**B**) tumor necrosis factor (TNF)-α secretion by the extracts of pepper leaves (PL) and pepper fruits (PF) in lipopolysaccharide (LPS)-stimulated RAW 264.7 cells. RAW 264.7 cells were treated with 100 (L), 200 (M), and 500 (H) µg/mL PL and PF extracts in the presence of 100 ng/mL LPS for 18 h. Statistical significance is based on the difference compared with CON(+) (** *p* < 0.01, *** *p* < 0.001).

The cytotoxicities of PL and PF on RAW 264.7 macrophages were measured by the MTS assay. PL and PF had no cytotoxicity of concern, with a cell viability of more than 80% on RAW 264.7 macrophages (Supplementary Figure S1). Compared to the LPS control group, 100 or 200 µg/mL of PL treatment could increase cell viability. Stimulation of RAW 264.7 macrophages by LPS increased the production of the inflammatory mediator NO [15]. The effects of PL and PF extracts on NO production in LPS-stimulated RAW 264.7 cells are shown in Figure 1. LPS-induced NO production was significantly decreased by the extracts of PL in a concentration-dependent manner. The PF extract significantly decreased NO production at concentrations of 200 and 500 µg/mL. The effects of PL and PF extracts on IL-6 and TNF-α production in LPS-induced RAW 264.7 cells are shown in Figure 2. The results revealed no significant decrease in the levels of the two inflammatory cytokines in the cells treated with the PF extracts. However, the PL extract (500 µg/mL) significantly decreased the production of both IL-6 and TNF-α in LPS-stimulated macrophages. 

### 3.3. Effect of the PL and Luteolin on ROS Production 

**Figure 3:**
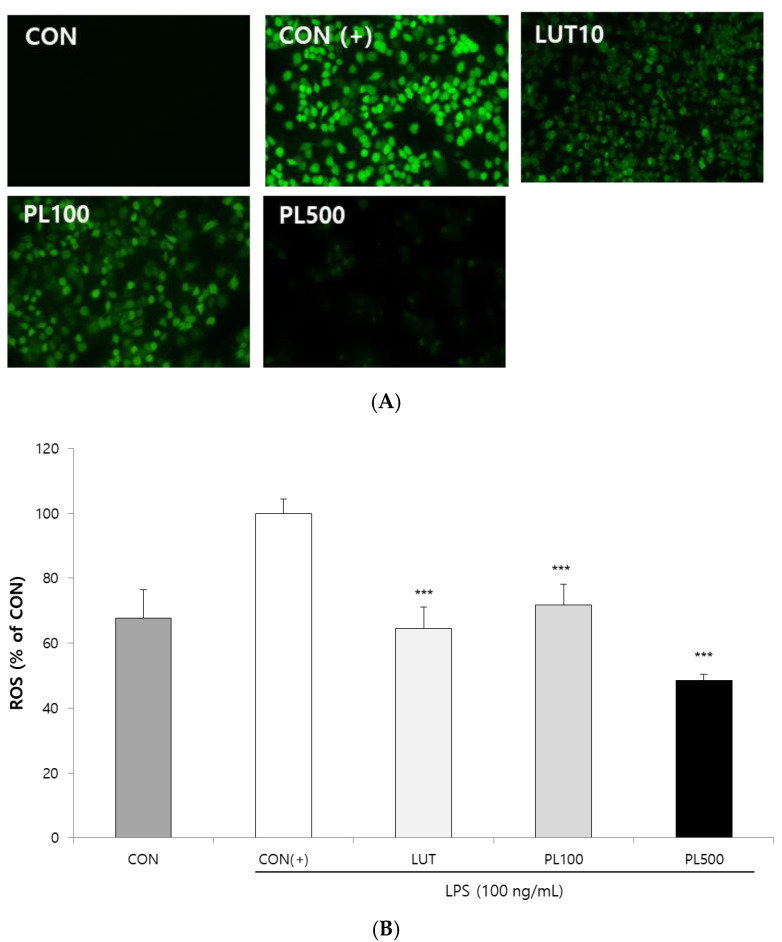
Pepper leaves (PL) extracts suppress reactive oxygen species (ROS) generation in lipopolysaccharide (LPS)-stimulated macrophages. (**A**) RAW 264.7 cells were treated with luteolin (LUT) 10 µM and various concentrations of PL extracts (100 and 500 µg/mL). Dichloro-dihydro-fluorescein diacetate (DCFH2-DA) was added to the culture medium at a final concentration of 50 µM. DCF fluorescence was imaged under a fluorescence microscope (100× magnification). (**B**) ROS inhibition rate, compared to control (+), was quantified by measuring the DCF fluorescence intensity. Statistical significance is based on the difference compared with CON (+) (*** *p* < 0.001).

LPS-induced ROS production was monitored in cells using DCFH_2_-DA fluorescence microscopic analysis. As shown in Figure 3, incubation of RAW 264.7 cells with LPS (100 ng/mL) significantly increased ROS generation, whereas co-treatment with the PL extract resulted in a significant dose-dependent reduction in ROS accumulation in LPS-induced RAW 264.7 cells. The ROS level was significantly decreased in the cells treated with PL extracts, similar to that observed with luteolin (10 µM) treatment. 

### 3.4. Flavonoid Content in the PL and PF Extracts 

Table 2 shows the flavonoid profile and content in the extracts of PL and PF. The pepper flavonoids were composed of *O*-glycosyl and C-glycosyl derivatives. Unlike *O*-glycosides, *C*-glycosides presented a specific pattern in which H_2_O (18 Da) was sequentially removed from the whole structure during MS fragmentation due to their strong carbon bonds. The total flavonoid content in PL was 2680.8 mg/100 g dry weight (DW), and the content of luteolin glycosides, which was identified as the major flavone in the PL, was 2608.9 mg/100 g DW. The total flavonoid and luteolin content in the PF was 1487.6 mg/100 g DW and 1371.1 mg/100 g DW, respectively. Except for apigenin glycosides, most flavonoids were present at higher abundance in PL than in PF. Luteolin glycosides accounted for 2.6% of the flavonoids PL and 1.4% of the flavonoids of PF. The 10 µM (approximately 2.9 µg/mL) of luteolin is similar to the content of 2.6 µg/mL of luteolin glycosides contained in 100 µg/mL of PL extracts. Luteolin 7-O-(2″-O-apiosyl) glucoside was present at a particularly large amount of 1472.6 mg/100 g DW in PL. 

## 4. Discussion

We investigated the antioxidant and anti-inflammatory effects of PL and PF in vitro, and their respective flavonoids contents. We found that the PL extracts were more effective in increasing radical scavenging activity and ameliorating inflammatory responses by inhibiting the LPS-induced production of NO, IL-6, and TNF-α in RAW 264.7 cells. In qualitative and quantitative analyses of flavonoids, the composition differed between PL and PF, in which the PL extracts had higher total flavonoids and, in particular, of luteolin contents than PF. 

The biological activities of pepper have mainly been studied on the fruit to date. Previous studies have reported the anti-inflammatory and antioxidant activities of red pepper stalk extracts, an agricultural waste product [16]. Shim and Han [17] reported the antioxidant activities of red pepper seed waste using various types of antioxidant assays. Most of the PL are also discarded because of their insufficient utilization, and there has been minimal research on these leaves. To the best of our knowledge, only two studies have reported the antioxidant activities of the leaves of red pepper cultivars and investigated pepper leaves-derived antioxidative compounds [7,18,19]. 

In our study, the PL extract more markedly scavenged DPPH and ABTS radicals than PF. Many oxidation products can cause cellular damage by interacting with biological materials; accordingly, oxidative stress has been linked to chronic diseases such as cancer, inflammation, and neurodegenerative diseases [20]. Dietary antioxidants were shown to have an inverse association with chronic disease development in a meta-analysis of prospective studies [21]. The present study confirmed that PL could be a promising source of effective dietary antioxidants. 

In addition, the PL extract more significantly inhibited LPS-induced NO and inflammatory cytokines production than PF. Treatment of macrophages with LPS, which is an endotoxin of gram-negative bacteria, results in various pathogenic effects such as the production of NO and inflammatory cytokines that regulate the inflammatory response [22]. The inflammatory cytokines such as IL-6 and TNF-α are particularly involved in pro-inflammatory responses. Moreover, LPS-activated macrophages induce the production of ROS, which were significantly reduced by PL. The PL extract showed comparable inhibition of LPS-induced ROS accumulation to that of luteolin in RAW 264.7 cells. Thus, our data confirmed that the antioxidant activity of the tested extracts was mediated by inhibiting ROS generation in macrophages. Because ROS generation is an important factor in the progression of the inflammatory process [23], these results indicate that PL can exert health benefits for inflammation-related diseases. Taken together, these findings suggest that PL is a better source of extremely useful antioxidant and anti-inflammatory compounds than PF according to the present experimental conditions.

The data on the flavonoid content in the PL and PF also provide strong evidence of these effects. Flavonoids are a class of secondary plant phenolics with significant antioxidant and anti-inflammatory properties [10,24]. Huang et al. [25] reported that the total flavonoid content in pepper was higher than that in Brassicaceae species such as cabbage, radish, and broccoli. Previous studies also reported that extracts of pepper stalk, placenta, pericarp, and seed are excellent sources of flavonoids [16,17]. The highest total flavonoid content was found in the pepper stalk. However, these studies analyzed all parts of the pepper except the leaves. In the present study, the content of total flavonoids in PL was 1.8 times higher than that in PF. 

Our results indicated that the composition and content of flavonoids in the PL and PF differed. The PL is the main source of flavonoids, and the luteolin content was the highest of all PL flavonoids at 91.4%, which was two times higher than that in PF. Luteolin 7-*O*-(2″-*O*-apiosyl)glucoside has been reported as a major flavone in *C. annuum* [4,26]. Similarly, our results showed that the content of luteolin 7-*O*-(2″-*O*-apiosyl)glucoside was the highest at 1472.6 mg/100 g DW in the PL. In addition, luteolin 7-*O*-apiosylmalonylglucoside, a major flavone in *C. annuum*, is a newly identified compound in PL, and further studies are required to confirm its structure. 

Luteolin 7-*O*-(2″-*O*-apiosyl)glucoside isolated from pepper fruits showed antioxidant and radioprotective effects [27]. It has been reported that luteolin in pepper has the highest antioxidant activity, followed by capsaicin and quercetin [28]. Luteolin (aglycone and its glycoside) from the pepper fruit showed the greatest antioxidant activity, followed by apigenin glycosides and apigenin [27]. Luteolin is a major contributor to anti-peroxidative activity compared with some flavonoids such as quercetin or kaempferol [29]. Our study also identified luteolin as major antioxidant compound in PL. ROS-reducing effects of PL extracts (100 µg/mL) containing approximately 2.6 µg/mL of luteolin glycosides was similar to that observed with luteolin treatment (10 µM, approximately 2.9 µg/mL). Luteolin inhibited the release of TNF-α and IL-6 in LPS-stimulated RAW 264.7 cells, which was more effective than that induced by quercetin and genistein [30]. Therefore, it is expected that PL with a high content of total flavonoids and luteolin would have stronger antioxidant and anti-inflammatory effects than those of PF, which was confirmed using the in vitro tests of the present study. 

The ripening stage or the variety of pepper plants is an important factor in the composition of bioactive compounds including flavonoids [31,32]. However, the present study is limited by not including differences in flavonoid content and antioxidant activity according to varieties or maturity. Further studies are needed to investigate antioxidant and anti-inflammatory activities at different ripening stages of the pepper plants in different varieties. Further investigation of the bioavailability of pepper extracts is also warranted.

## 5. Conclusions

We investigated the flavonoid content of *C. annuum* and its antioxidant and anti-inflammatory effects in vitro. We found that the PL extracts were more effective in increasing radical scavenging activity and ameliorating inflammatory responses by inhibiting the LPS-induced production of NO, IL-6, and TNF-α in RAW 264.7 cells. In qualitative and quantitative analyses of flavonoids, the composition differed between PL and PF. PL extracts had higher total flavonoids and luteolin contents than PF. We show that luteolin-enriched PL is a healthy food that may effectively alleviate inflammatory responses and oxidative stress.

## Figures and Tables

**Table 1:** DPPH and ABTS·^+^ assays of radical scavenging activity of pepper leaves and pepper fruits.

**Samples**	**DPPH radical scavenging activity (%)**			
	**EC**_**50**_ ** (µg/mL)**	**100 µg/mL**	**200 µg/mL**	**500 µg/mL**
Pepper leaf	159.1 ± 8.4	42.8 ± 0.5	61.4 ± 2.1	90.1 ± 1.0
Pepper fruit	653.4 ± 30.4	26.6 ± 2.0	32.1 ± 1.4	42.7 ± 0.7
Luteolin	2.1 ± 0.1	-	-	-
Ascorbic acid	6.4 ± 0.9	-	-	-
**Samples**	**ABTS·^+^ radical scavenging activity (%)**			
	**E** **C_50_ (µg/mL)**	**100 µg/mL**	**200 µg/mL**	**500 µg/mL**
Pepper leaf	157.6 ± 14.6	45.6 ± 2.0	71.7 ± 2.8	92.1 ± 4.1
Pepper fruit	357.1 ± 21.7	25.9 ± 1.4	37.7 ± 1.1	59.5 ± 8.1
Luteolin	3.3 ± 0.2	-	-	-
Ascorbic acid	23.7 ± 0.1	-	-	-

**Table 2:** Characterization of individual flavonoids in pepper leaves (PL) and pepper fruits (PF) ^1^.

Classes	Sub-Classes	Individual Flavonoids	ESI(+)-QToF–MS	Contents (mg/100g Dry Weight)	
			Fragmentation of [M + H]^+^ (*m/z*)	PL	PF
Flavones	Apigenin	Apigenin 6,8-di-C-glucoside (vicenin-2)	617, 595, 577, 559, 523	ND ^2^	38.7 ± 2.6
		Apigenin 6-*C*-arabinoside-8-*C*-glucoside (isoschaftoside) ^3^	587, 565, 547, 529, 335	ND	24.1 ± 1.7
		Apigenin 6-*C*-glucoside-8-*C*-arabinoside (schaftoside) ^3^	587, 565, 547, 529, 445, 335	ND	18.4 ± 0.8
		Apigenin 7-*O*-glucoside (cosmosiin) ^3^	455, 433, 271	5.9 ± 0.9	ND
	Sub total			5.9 ± 0.9	81.2 ± 5.1
	Luteolin	Luteolin 6,8-di-*C*-glucoside (lucenin-2)	633, 611, 593, 575, 401, 373	ND	13.0 ± 0.9
		Luteolin 6-*C*-arabinoside-8-*C*-glucoside	603, 581, 563, 545, 527, 371	ND	3.1 ± 0.3
		Luteolin 6-*C*-glucoside-8-*C*-arabinoside (carlinoside)	603, 581, 563, 545, 461, 371	ND	12.9 ± 0.9
		Luteolin 8-*C*-glucoside (orientin) ^3^	471, 449, 431, 413, 329	ND	60.7 ± 2.2
		Luteolin 6-*C*-glucoside (isoorientin) ^3^	471, 449, 431, 329	ND	16.6 ± 0.4
		Luteolin 7-*O*-(2″-*O*-apiosyl)glucoside	603, 581, 449, 287	1472.6 ± 107.2	98.2 ± 2.3
		Luteolin 7-*O*-apiosylmalonylglucoside ^4^	689, 667, 535, 287	1136.3 ± 18.5	1166.7 ± 63.1
	Sub total			2608.9 ± 116.2	1371.1 ± 61.7
Flavonols	Quercetin	Quercetin 3-*O*-rhamnoside-7-*O*-glucoside	633, 611, 465, 449, 303	ND	ND
		Quercetin 3-*O*-glucoside (isoquercitrin) ^3^	487, 465, 303	ND	24.3 ± 0.8
		Quercetin 3-*O*-rhamnoside (quercitrin) ^3^	471, 449, 303	66.0 ± 1.6	10.9 ± 0.3
	Sub total			66.0 ± 1.6	35.3 ± 1.1
TOTAL				2680.8 ± 117.2	1487.6 ± 58.8

^1^ All samples were analyzed in positive ESI ionization mode (m/z, [M + H]+) using UPLC–DAD–QToF–MS; [M + Na]+ adduct ion presented. Each of the compounds was identified by comparing elution order, UV spectra, and mass fragmentation with the in-house library constructed from literature sources. Bold font indicates parent ion ([M + H]+) of flavonoid structures. Each value calculated as means ± SD (*n* = 3) using galangin as the internal standard. ^2^ ND, not detected. ^3^ Further confirmed in comparison with authentic standards. ^4^ New compound tentatively identified.

## Supplementary Materials

The following are available online at https://www.mdpi.com/2076-3921/9/10/986/s1, Figure S1: The cytotoxicity of pepper leaf (PL) and pepper fruit (PF) extracts on RAW 264.7 macrophages was measured by MTS assay.
