# Supplementary material for: Antioxidant and Anti-Inflammatory Activities in Relation to the Flavonoids Composition of Pepper (Capsicum annuum L.)
Source: Antioxidants (Basel). 2020 Oct 13;9(10):986. doi: 10.3390/antiox9100986 (PMC7602036; doi:10.3390/antiox9100986)
Supplement: Supplementary file 1 [file antioxidants-09-00986-s001.pdf]

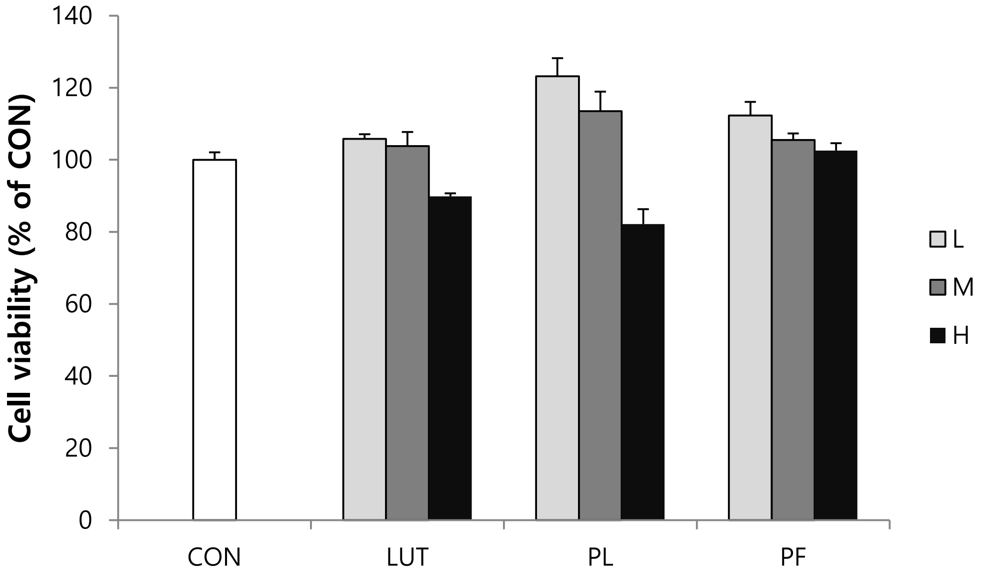

Supplementary Figure S1. The cytotoxicity of pepper leaves (PL) and pepper fruits (PF) extracts on RAW 264.7 macrophages was measured by MTS assay. RAW 264.7 cells were treated with 100 (L), 200 (M), and 500 (H) µg/mL of PL and PF extracts or 2 (L), 4 (M), and 10 (H) µM of luteolin (LUT) in the presence of 100 ng/mL LPS for 18 h.
